# Artificial Intelligence-Powered Molecular Docking and Steered Molecular Dynamics for Accurate scFv Selection of Anti-CD30 Chimeric Antigen Receptors

**DOI:** 10.3390/ijms25137231

**Published:** 2024-06-30

**Authors:** Nico Martarelli, Michela Capurro, Gizem Mansour, Ramina Vossoughi Jahromi, Arianna Stella, Roberta Rossi, Emanuele Longetti, Barbara Bigerna, Marco Gentili, Ariele Rosseto, Riccardo Rossi, Chiara Cencini, Carla Emiliani, Sabata Martino, Marten Beeg, Marco Gobbi, Enrico Tiacci, Brunangelo Falini, Francesco Morena, Vincenzo Maria Perriello

**Affiliations:** 1 Institute of Hematology and Center for Hemato-Oncology Research, University of Perugia and Santa Maria della Misericordia Hospital, 06132 Perugia, Italy; nico.martarelli@gmail.com (N.M.); michela.capurro@libero.it (M.C.); vossoughi.r@gmail.com (R.V.J.); arianna.stella89@gmail.com (A.S.); roberta.rossi78@virgilio.it (R.R.); emanuelelongetti@gmail.com (E.L.); bigernab@gmail.com (B.B.); marcogentili1988@hotmail.it (M.G.); rosseto.ariele@gmail.com (A.R.); riccardo82rossi@gmail.com (R.R.); enrico.tiacci@unipg.it (E.T.); brunangelo.falini@unipg.it (B.F.); 2 Istituto di Ricerche Farmacologiche Mario Negri IRCCS, Via Mario Negri 2, 20156 Milano, Italy; gizem.erol@helmholtz-munich.de (G.M.); marten.beeg@marionegri.it (M.B.); marco.gobbi@marionegri.it (M.G.); 3 Department of Chemistry, Biology, and Biotechnologies, Via del Giochetto, University of Perugia, 06122 Perugia, Italy; chiara.cencini@studenti.unipg.it (C.C.); carla.emiliani@unipg.it (C.E.); sabata.martino@unipg.it (S.M.)

**Keywords:** chimeric antigen T cells (CAR-T), artificial intelligence (AI), molecular docking (MD), steered molecular dynamics (SMD), coarse grained umbrella sampling (CG-US), Hodgkin’s lymphoma, CD30

## Abstract

Chimeric antigen receptor (CAR) T cells represent a revolutionary immunotherapy that allows specific tumor recognition by a unique single-chain fragment variable (scFv) derived from monoclonal antibodies (mAbs). scFv selection is consequently a fundamental step for CAR construction, to ensure accurate and effective CAR signaling toward tumor antigen binding. However, conventional in vitro and in vivo biological approaches to compare different scFv-derived CARs are expensive and labor-intensive. With the aim to predict the finest scFv binding before CAR-T cell engineering, we performed artificial intelligence (AI)-guided molecular docking and steered molecular dynamics analysis of different anti-CD30 mAb clones. Virtual computational scFv screening showed comparable results to surface plasmon resonance (SPR) and functional CAR-T cell in vitro and in vivo assays, respectively, in terms of binding capacity and anti-tumor efficacy. The proposed fast and low-cost in silico analysis has the potential to advance the development of novel CAR constructs, with a substantial impact on reducing time, costs, and the need for laboratory animal use.

## 1. Introduction

Chimeric antigen receptor (CAR) T cells represent a major breakthrough in cancer immunotherapy in which immune effector cells are genetically engineered by a synthetic molecule to recognize surface antigens for tumor cell killing [1]. CARs combine an antigen-binding site with T cell activation and co-stimulation domains, allowing a specific MHC-independent immune response and prolonged immune memory [2]. The antigen-binding domain mainly derives from variable heavy (VH) and light (VL) chains of monoclonal antibodies (mAbs) connected by a flexible linker to assemble a single-chain variable fragment (scFv). Appropriate identification of mAbs-derived scFv is therefore crucial during CAR design to ensure therapeutic anti-tumor activity [3,4], considering their unique ability to bind target antigens with specificity and affinity. Based on the mAbs source, selection of high-binding activity scFv for CAR construction is commonly a result of conventional expansive or time-consuming techniques such as 10^8^–10^10^ phage display library screenings [5] or in vitro and in vivo activity comparison of different CAR-T cells expressing available scFv sequences [6]. The development of novel scFv screening methods, improving faster CAR design and at a lower cost, is therefore of extreme importance.

Recently, artificial intelligence (AI) techniques have allowed accurate predictions of 3D protein structures, opening a new era of in silico mAb/antigen complex investigations, and increasing the reliability of computational docking techniques such as molecular dynamics (MD) [7]. MD simulations reveal atomic-level insights into antigen–antibody recognition [8], providing important data toward binding interactions [9]. Consequently, AI-aided MD approaches could offer the opportunity to advance CAR design as compared to conventional scFv screenings through (i) virtual scFv selection from available mAbs sequences before CAR engineering, based on affinity prediction and epitope-binding identification; (ii) containing costs by reducing waste of laboratory reagents and refining animal use; and (iii) optimizing researchers’ workforce time while running the proposed computational analysis.

In this study, we employed innovative AI information-driven docking and coarse-grained steered molecular dynamics to investigate the molecular interactions between different anti-CD30 mAb clones and their target antigen. CD30 is an excellent candidate for high-affinity scFv-based therapies due to its restricted expression on Hodgkin Reed–Sternberg (HRS) malignant cells and limited expression on healthy cells [10]. The goal was to predict the optimal anti-CD30 scFv for CAR construction targeting CD30 in classic Hodgkin’s Lymphoma (cHL), where the impact of CAR-T cells has been limited thus far [11]. Our proposed comprehensive in silico analysis was compared to surface plasmon resonance (SPR) and conventional in vitro/in vivo CAR validation to prove consistency and reliability. This integrative approach not only showcases the potential of AI-driven techniques to advance CAR design but also underscores the importance of computational methods in expediting therapeutic advancements.

## 2. Results

### 2.1. Generation and Validation of Novel Anti-CD30 mAbs

**Figure 1:**
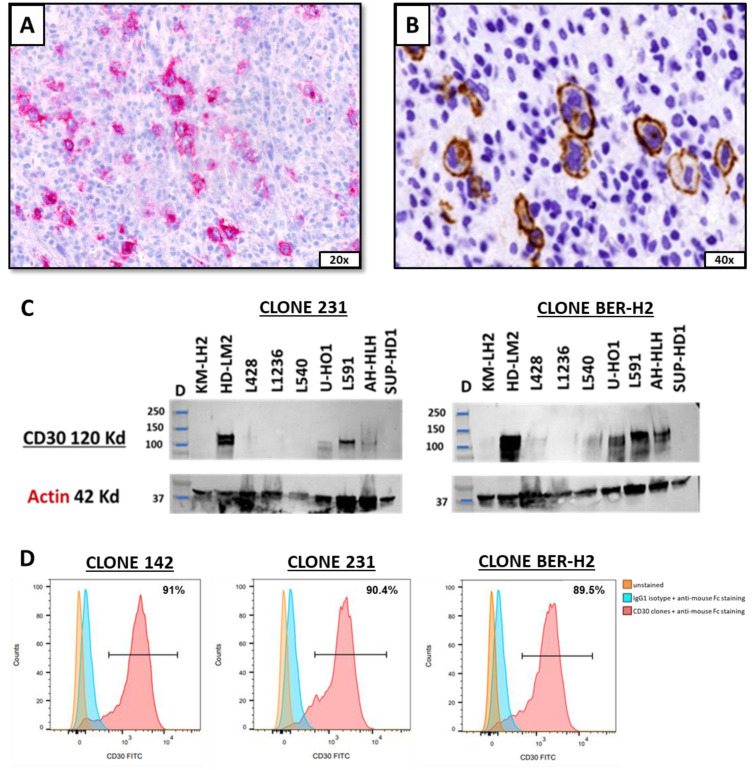
Screening and validation of novel anti-CD30 mAbs: (**A**) Human tonsil immunohistochemically stained by selected novel anti-CD30 mAb, highlighting large T lymphocytes in follicular germinal center. (**B**) Newly developed anti-CD30 mAb specifically staining neoplastic cells in cHL in paraffin-embedded sample. (**C**) Western Blot analysis showing a 120 kDa band in cHL cell lines lysates and predicted to be CD30 molecular weight, detected by the designated mAb clone 231, similar to the already validated anti-CD30 BER-H2 clone. (**D**) Flow cytometry analysis showing equal positive CD30 expression between anti-CD30 mAb clones on the AH-HLH 200 cHL cell line, as compared to controls.

Novel anti-CD30 mAbs were produced through hybridoma technology after BALB/C mice immunization with a 392 amino acid his-conjugated soluble peptide, corresponding to the CD30 extracellular portion. Hybridoma colonies supernatants were initially screened by immunohistochemistry (IHC) on frozen sections of human tonsil. Predicted anti-CD30 clones were identified upon selectively staining a subpopulation of large activated T lymphocytes predominantly located around the mantle areas of the B follicles and, sometimes, inside the germinal centers (Figure 1A). Two distinct anti-CD30 mAbs clones were further selected for strong and specific reactivity against Hodgkin and Reed–Sternberg tumor cells on Hodgkin lymphoma (Figure 1B). Those anti-CD30 clones screened by IHC, named 142 and 231, showed reactivity patterns similar to an already-known anti-CD30 mAb (BER-H2), previously investigated as an immunotherapeutic drug in r/r cHL patients [12].

To evaluate the specificity of predicted anti-CD30 clones, Western blotting and flow cytometry analysis were performed on several cHL cell lines. CD30 protein migration in Western blotting was confirmed by comparing novel selected anti-CD30 mAb with the anti-CD30 BER-H2 clone. A band of 120 kDa (corresponding to the molecular weight of CD30) was detected in the lane of cHL lines lysates strongly expressing CD30. A similar expression profile was detected by BER-H2 anti-CD30 mAb (Figure 1C).

The recognition of the CD30 extracellular portion was evaluated by flow cytometry on the human Hodgkin’s lymphoma CD30+ AH-HLH 200 cell line, showing an equal CD30 positivity among all newly generated and BER-H2 anti-CD30 clones (Figure 1D). Hybridoma cells from each clone were subsequently subcloned four times to ensure monoclonality and hereafter used to sequence the antibody heavy- and light-chain variable domains (complementarity-determining regions CDRs), characteristic for specific antigen binding.

### 2.2. AI-Guided 3D Structure Prediction of Anti-CD30 mAbs/CD30 Antigen-Binding Interaction

**Figure 2:**
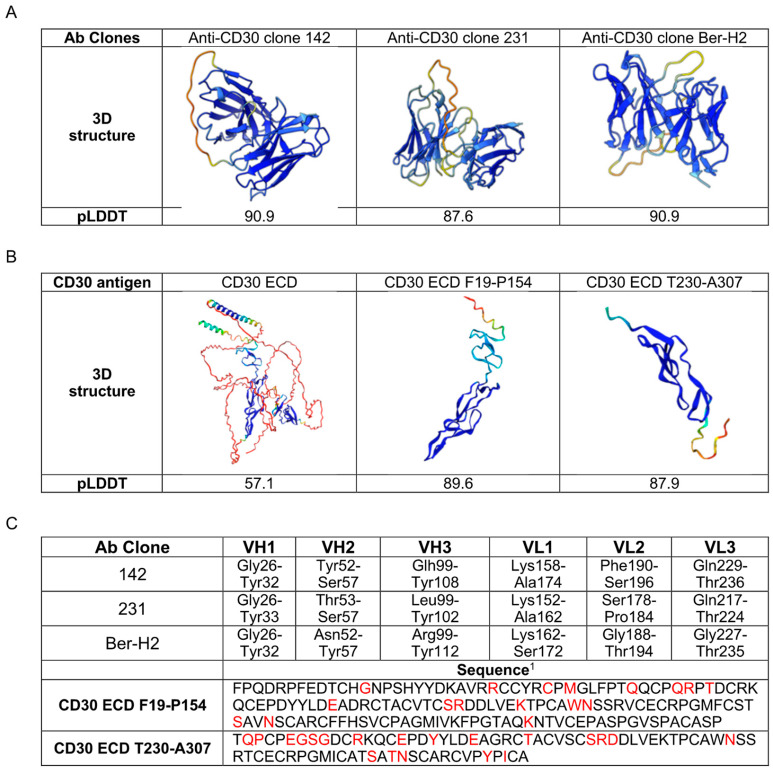
Predicted 3D structures of antibody clones and antigen, and predicted paratope and epitope: (**A**) Three-dimensional structures of antibody clones and their pLDDT score. (**B**) Three-dimensional structures of total extracellular CD30 antigen (residues 1-392), CD30 F19-P154 antigen, and CD30 T230-A307 antigen, with their pLDDT score. (**A**,**B**) The pLDDT scores are visually represented in AlphaFold models through color-coded intervals. Scores above 90 indicate very high confidence (dark blue), between 70 and 90 represent good confidence (green to cyan), between 50 and 70 reflect low confidence (yellow), and below 50 indicate very low confidence (orange). (**C**) Prediction of VH and VL chain of different clones, and epitope prediction of the two CD30 ECD regions. Predicted residues as marked in red.

To evaluate anti-CD30 mAbs binding capacity toward CD30 antigen, we used a molecular docking technique based on AI-driven protein structures. First, we performed the prediction of the 3D structure of anti-CD30 mAb clones with identical scFv orientation VH-VL and the CD30 antigen, using the Alphafold2 [13] deep learning-based method. The predicted 3D structures generated by AlphaFold2 are presented alongside the predicted local distance difference test (pLDDT) in Figure 2A,B. The pLDDT score serves as a robust indicator of the accuracy of amino acid distances within protein structures, providing an overall assessment of structure quality and identifying regions of high and low confidence. Based on the pLDDT score, the 3D structures of all anti-CD30 clones exhibit around 90 score values indicating the highest confidence level (Figure 2A). The region with low confidence indicated by pLDDT (yellow/orange), refers to the scFv linker portion between variable heavy (VH) and light (VL) chains, in common with all mAb clones. Since no experimental structure of the CD30 ECD was available in the PDB database, we utilized AlphaFold2 to generate a structural model. The total length of CD30 ECD was predicted, where the pLDDT scores indicated superior structural quality with higher confidence levels in regions F19-P154 and T230-A307 (Figure 2B). A focused analysis in these regions allowed us to conduct further in-depth analysis and exploration of almost all CD30 extracellular epitopes [14].

Two machine learning methods were used to pinpoint the specific regions on both the anti-CD30 antibodies and the CD30 antigen that likely mediate their interaction. Antibody-binding sites were predicted using ProABC-2, a sophisticated method employing a convolutional neural network within a random forest framework. For the antigen, potential binding regions were identified using DiscoTope-3.0, an XGBoost model that leverages the three-dimensional structure of CD30 predicted by AlphaFold2. The findings from the predictive models are depicted in Figure 2C. These results highlight the specific residues from both the variable heavy chain (VH-CDR1, VH-CDR2, and VH-CDR3) and the light chain (VL-CDR1, VL-CDR2, and VL-CDR3) of the anti-CD30 monoclonal antibodies. For the CD30 antigen, the likely binding regions are highlighted in red, spanning residues F19-P154 and T230-A307 of the extracellular domain (ECD). Machine learning-predicted hypervariable loop and epitope residues were utilized to conduct subsequent molecular docking with the aim of identifying the best scFv for anti-CD30 CAR construction.

### 2.3. Modeling CD30 Antibody–Antigen Complexes by AI Information-Driven Docking

The HADDOCK webserver was used to conduct AI information-driven docking for modeling the interaction of anti-CD30 mAb clones and CD30 antigen in the 3D-predicted regions F19-P154 and T230-A307. The results demonstrated that all three clones (142, 231, and Ber-H2) were effectively bound to the CD30 antigen (Table S1), with energy scores ranging from −98 to −61 for the top 1 cluster in both CD30 regions. In all cases, the CD30 F19-P154 region exhibits lower HADDOCK scores compared to the CD30 T230-A307 region, suggesting a potentially stronger binding affinity for the F19-P154 region in the docking complexes. Therefore, due to the consistently lower HADDOCK scores across all clones and the implication of potentially stronger binding affinity, we selected the CD30 F19-P154 region for further analysis.

**Figure 3:**
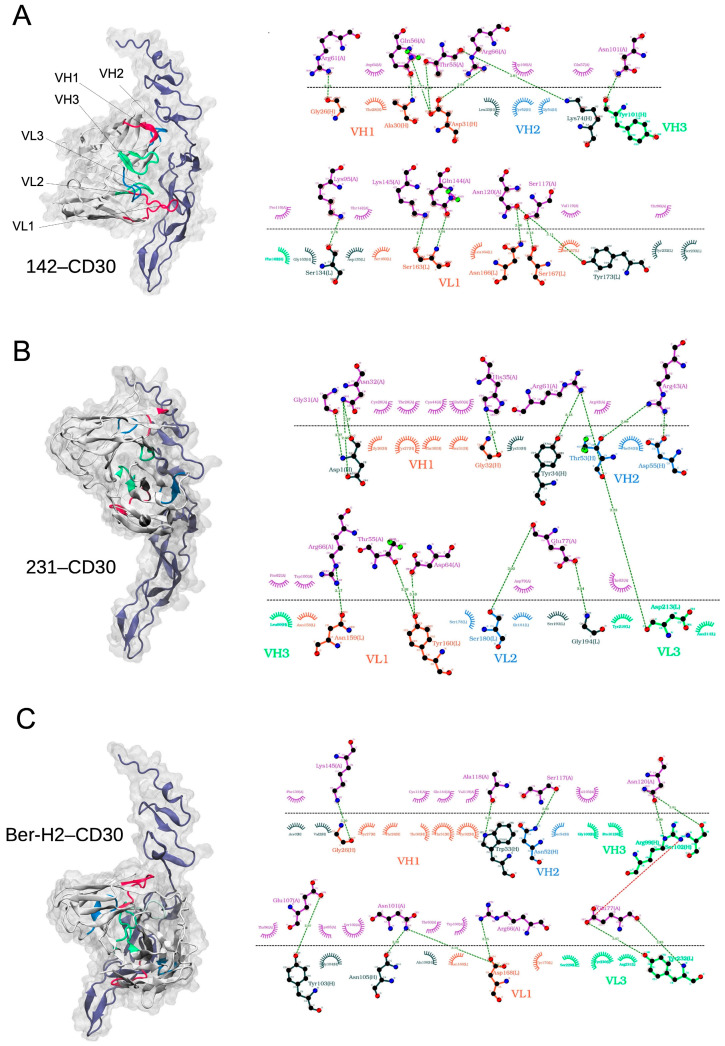
Docking model interaction between CD30 and the three different antibody clones: Contact surface of CD30 in complex with clone 142 (**A**), clone 231 (**B**), and clone Ber-H2 (**C**), and corresponding two-dimensional diagram of the interaction. The docking model indicated 13 intermolecular hydrogen bonds in the 142–CD30 complex, 13 intermolecular hydrogen bonds in the 231–CD30 complex, and 11 intermolecular hydrogen bonds in the Ber-H2–CD30 complex.

Hydrogen bonds play major roles in the binding interaction between CD30 F19-P154 antigen region and anti-CD30 mAb clones. CD30 might form hydrogen bonds (i) with the sites of Gly26, Ala30, Asp31 of VH-CDR1; Tyr101 of VH-CDR3, and Ser163, Asn166, Ser167 of VL on clone 142 (Figure 3A); (ii) with the sites of Gly32 of VH-CDR1; Thr53 and Asp55 of VH-CDR2; Asn159 and Tyr160 of VL-CDR1, and Ser180 of VL-CDR2, on clone 231 (Figure 3B); and (iii) with the sites of Gly26 of VH-CDR1; Asn52 of VH-CDR2; Arg99 and Ser102 of VH-CDR3; Asp168 of VL-CDR1, and Tyr232 of VL-CDR3, on clone Ber-H2 (Figure 3C).

In the docking model, we observed the formation of 13 intermolecular hydrogen bonds in the 142–CD30 complex, 13 intermolecular hydrogen bonds in the 231–CD30 complex, and 11 intermolecular hydrogen bonds in the Ber-H2–CD30 complex, for the top 1 cluster (as shown in Table S1). Thus, the anti-CD30 mAb clones 142, 231, and Ber-H2 form stable and energetically favorable complexes with CD30 antigen, supported by the observed intermolecular hydrogen bond formations in the docking models.

Overall, these findings provide insights into the interaction sites and binding affinities of clones 142, 231, and Ber-H2 with the CD30 antigen, highlighting the preference for the CD30 F19-P154 region in terms of stronger binding based on the lower HADDOCK scores.

### 2.4. Molecular Dynamics Simulation Identify the Most Stable CD30 Antibody–Antigen Complex

The antibody–antigen interaction of the different complexes was probed using an AI information-driven HADDOCK molecular docking protocol. However, the dynamics of antigens and antibodies were not considered in the docking simulations, and the number of trial docking poses was restricted. To address this limitation, we performed 100 ns all-atomistic molecular dynamics simulations on the top 3 clusters for each complex. This extended simulation not only provided insights into the dynamics of antigens and antibodies but also served as an additional assessment of the robustness and reliability of the docking results. The root mean square deviations (RMSDs) were subsequently analyzed to evaluate the balance of trajectory.

**Figure 4:**
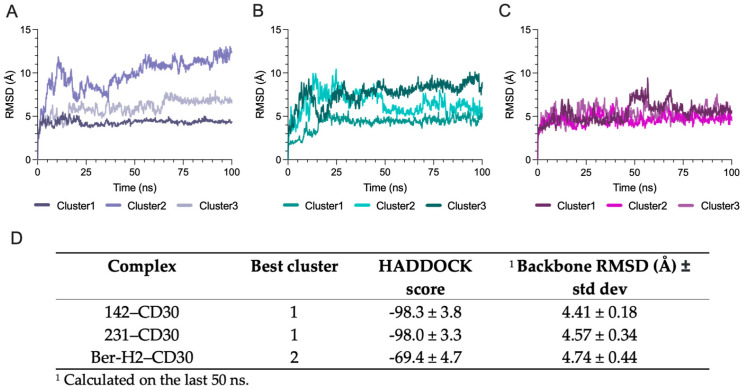
Atomistic dynamic simulations (100 ns) of the top 3 clusters for each Ab–Ag complex: (**A**) Backbone RMSD of 142–CD30 complex. (**B**) Backbone RMSD of 231–CD30 complex. (**C**) Backbone RMSD of Ber-H2–CD30 complex. (**D**) Summary table of the most stable cluster selected for each complex.

As depicted in Figure 4A, the 142–CD30 system rapidly reached the equilibrium state in cluster 1, while cluster 2 exhibited a higher RMSD value and approached equilibrium after 65 ns. Cluster 3 showed the highest RMSD value and did not converge to the equilibrium state. In the 231–CD30 system, equilibrium was reached after approximately 25 ns in all clusters. However, clusters 2 and 3 were the most unstable, as indicated by RMSD fluctuations, suggesting ongoing conformational changes. In contrast, cluster 1 was the most stable, displaying the lowest RMSD (Figure 4B). In the Ber-H2–CD30 system, cluster 2 demonstrated the highest stability, with a lower RMSD value and approaching equilibrium after 25 ns. Conversely, clusters 1 and 3 exhibited greater instability, characterized by higher fluctuations in RMSD, suggesting a tendency for conformational changes (Figure 4C). Cluster 1 for the 142–CD30 complex, cluster 1 for the 231-CD30 complex, and cluster 2 for the Ber-H2–CD30 complex were identified as the most stable. The RMSD analysis of the antibody–antigen complexes revealed that over the 100 ns MD time course, none of the antigens spontaneously dissociated from the core of the antibody structure. Additionally, there were no observable conformational changes in the antigens of the selected clusters, which exhibited the most stable RMSD (Figure 4D). These clusters were selected for the computational study of binding affinity.

### 2.5. Coarse-Grained (CG) Steered Molecular Dynamics (SMD) Simulations Provide Insights into the Relative Binding Strengths of CD30 mAb-CD30 Antigen Pairs

Steered molecular dynamics simulations have proven effective in investigating various processes, such as the binding of proteins and ligands, interactions between proteins, protein–nucleic acid, and lipids membranes [15,16,17,18,19]. By applying external forces to manipulate the system, SMD can accelerate rare events and overcome the time scale limitations inherent in conventional molecular dynamics simulations, providing valuable insights into phenomena that would otherwise be challenging to observe.

**Figure 5:**
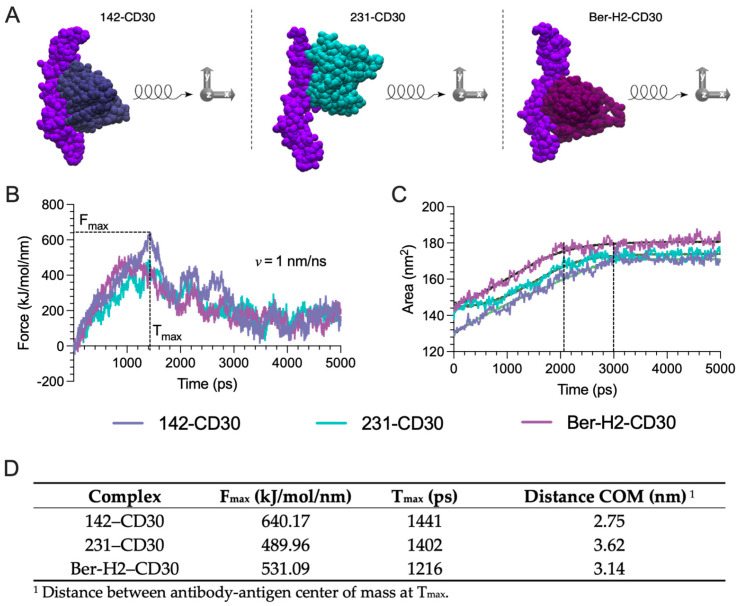
Pulling Simulations: (**A**) Schematic representation of force applied to XYZ-direction vectors connecting the antibody–antigen interface for all simulated systems. (**B**) Force profile to dissociate the antigen CD30 from three different antibodies: 142–CD30, 231–CD30, and Ber-H2–CD30 as a function of time. A constant pulling velocity of 1 nm/ns was applied. (**C**) Plots of the total solvent accessible surface area (SASA) of the complexes over the SMD simulation time. (**D**) Summary data of SMD simulations, reporting rupture force (F_max_), rupture time (T_max_), and center-of-mass distance (COM).

Here, we carried out coarse-grained SMD simulations (CG-SMD) to pull 142, 231, and Ber-H2 clones from the binding region of CD30 antigen as well as pulling CD30 antigen from the binding region of 142, 231, and Ber-H2 clones. By pulling on the COM of the antibody, force builds up until a breaking point is reached, at which time critical interactions are disrupted, allowing the antibody to dissociate from the CD30 antigen structure. We applied an external pull force of 1000 kJ mol perpendicular to the antibody–antigen interface and a soft constrain force (100 kJ mol) on the antigen’s backbone to prevent the antigen conformation from undergoing unfolding or being pulled (drifting) with the antibody during the pull process. The pulling direction was in the XYZ-direction with respect to the vectors connecting COMs of the antigen and antibody (Figure 5A).

In the SMD simulations, the maximum force (Fmax) was reached before the disruption of interchain backbone hydrogen bonds between each antibody and its CD30 antigen-binding residues. The force–time curves, generated at a pulling velocity of 1 nm/ns, reveal that the 142-antibody clone establishes the most resilient complex with CD30. This is evidenced by the necessity of the highest peak force, reaching 640.17 kJ/mol/nm for unbinding, implying the formation of the most stable association with CD30. In comparison, clones 231 and Ber-H2 dissociated from the CD30 antigen at lower maximum forces of 489.96 kJ/mol/nm and 531.09 kJ/mol/nm, respectively. The lower Fmax values suggest that these two antibody clones form weaker interactions with the CD30 antigen compared to the 142 clones. Overall, the peak force measurements imply that the stability of the antibody–antigen complexes follow the order 142–CD30 > Ber-H2–CD30 > 231–CD30 (Figure 5B). The binding times T_max_ of 142–CD30 and 231–CD30 are quite similar, but slightly longer than Ber-H2–CD30, with a COM distance of ≅2.75 nm, 3.62 nm, and 3.14 nm, respectively. Moreover, disruption of native packing at T_max_ allowed additional water molecules to interact with the antibody. The solvent-accessible surface area (SASA) increases as the complexes dissociate and the buried surface area becomes exposed (Figure 5C). Specifically, a rapid rise in SASA is observed for 231–CD30 and Ber-H2–CD30, corresponding to their relatively lower stability in the simulation. Approximately 2 ns into the simulation, both the 231–CD30 and Ber-H2–CD30 complexes reach a plateau, exhibiting solvent-exposed areas of about 172 nm^2^ and 180 nm^2^, respectively. This suggests that these complexes have transitioned to a complete unbinding state, fully exposed to the solvent. In contrast, for the 142–CD30 complex, SASA shows a gradual increase, reaching a plateau after 3 ns with an exposed area of approximately 171 nm^2^. This observation implies that a more prolonged application of force and time is required to achieve the unbinding state, aligning with its higher stability.

In summary, the SMD simulations and SASA calculations demonstrate that clone 142 forms the most stable complex with the CD30 antigen compared with 231–CD30 and Ber-H2–CD30 complexes, providing insights into the relative binding strengths of antibody–antigen pairs.

### 2.6. CD30 mAb Binding Affinity Estimation through Umbrella Sampling (US) Simulations Are Comparable to Surface Plasmon Resonance (SPR)

To estimate the binding free energy (∆G-bind) for a specific event along the reaction coordinate corresponding to the XYZ-axis, we used equilibrium methods such as umbrella sampling and weighted histogram analysis (WHAM). These methods reconstruct ensemble properties from multiple trajectories to account for diverse dissociation routes. By utilizing 72 sampling windows (each separated by 0.5 nm) along the reaction coordinate, one-dimensional potential of mean force (PMF) curves were generated for each complex, enabling the calculation of ∆G binding.

**Figure 6:**
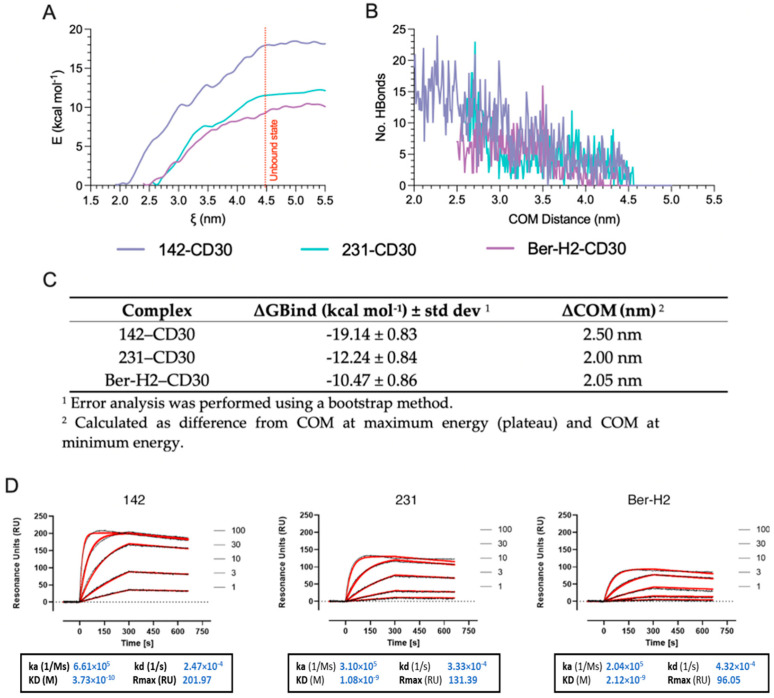
Comparison between US analysis and SPR: (**A**) Potential of mean force profiles (kcal mol^−1^) for the antibody–antigen complexes as a function of distance (nm) during the separation. The red dotted line represents the distance at the unbound state. Each PMF profile shown above is shifted so that the average PMF value in the bound state is 0 kcal mol^−1^. (**B**) Total number of hydrogen bonds as a function of separation distance (nm), formed between the antigen and the antibody during the unbinding event. (**C**) Summary table for each complex of ∆GBind and ∆COM. (**D**) Sensorgrams obtained by injecting five concentrations of CD30 (1–100 nM) for five min over different mAbs pre-captured on the SPR sensor chip. The sensorgrams (black lines) were obtained after subtraction of the non-specific signal obtained on the reference surface, and thus represent the specific binding of CD30 to the mAbs. For each mAb, sensorgrams were globally fitted with a 1:1 interaction model and the fitting is shown in red. ka: association rate constant; kd: dissociation rate constants; KD: equilibrium dissociation constant, Rmax: maximum binding capacity.

The analysis of the resulting PMF curves showed that the energy minimum occurred at a COM distance of 1.97 nm, 2.66 nm, and 2.43 nm for the 142–CD30, 231–CD30, and Ber-H2-CD30, respectively (Figure 6A). As antibody–antigen interactions decrease in the unbound state, the potential of mean force profiles for each complex tends to approach a plateau and full convergence. After 4.5 nm for the 142–CD30, for the 231–CD30, and for Ber-H2-CD30 (Figure 6A, dotted line), the antigen–antibody complexes are in the unbound state. This state is primarily a result of the loss of hydrogen bonds between the surface of the antibody and the antigen at the interacting interface (Figure 6B), confirming the COM distance in the unbound state observed in the PMF curve. During the binding time, the 142–CD30 complex exhibited a higher number and higher interaction time of hydrogen bonds, in contrast to the 231–CD30 and Ber-H2–CD30 complexes, which had fewer hydrogen bonds and a rapid drop in their number (Figure 6B). Overall, the data indicate that the 142–CD30 complex showed the maximum energy (−19.14 kcal mol^−1^), followed by the 231–CD30 (−12.24 kcal mol^−1^) and Ber-H2–CD30 (−10.47 kcal mol^−1^) complexes (Figure 6C).

Surface plasmon resonance (SPR) was exploited for a parallel comparison of the binding properties of the different anti-CD30 mAb clones, immobilized on different lanes of the chip using anti-mouse IgGs to capture the antibodies in hybridoma supernatants on different lanes of the chip 18. After rotation of the flow channels, different concentrations of the antigen (CD30) could be flowed simultaneously on all the immobilized antibodies, allowing a reliable determination of the binding constants. Figure 6D shows the specific, concentration-dependent binding of CD30 to different pre-captured mAbs on the SPR sensor chip. For all anti-CD30 mAbs, the sensorgrams obtained at the different CD30 concentrations (black lines, Figure 6D) could be globally fitted by a 1:1 interaction model (red lines, Figure 6D).

Clone 142 was confirmed to exhibit the highest binding affinity for CD30, with a KD value of 0.37 nM. Clones 231 and Ber-H2 showed a KD of 1–2 nM, mainly because of a 2–3-fold slower association rate compared to clone 142.

### 2.7. Anti-Tumor Activity of Newly Generated CD30 CAR-T Cells Confirm AI-Guide In Silico Predictions

To evaluate the effector functions of newly generated CD28-based second-generation anti-CD30 CAR-T cells differing only for scFv, we initially performed three different in vitro assays in order to assess their killing profile, proliferation rate, and cytokine release after encountering the CD30 target antigen expressed on the HD-LM2 Hodgkin’s Lymphoma cell line.

**Figure 7:**
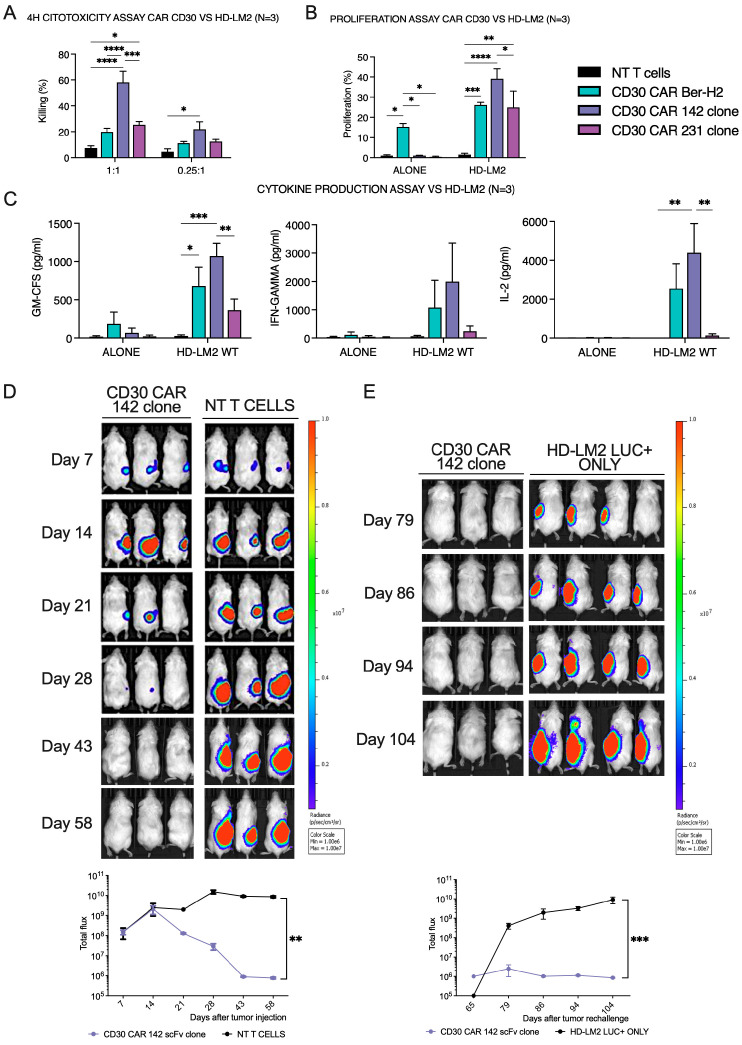
Anti-tumor activity of anti-CD30 CAR-T cells derived from clones 142, 231, and BER-H2 mAbs: CD30 CAR-T cells derived from clone 142 scFv shown the highest in vitro activity against HD-LM2 cHL cell lines by means of cytotoxicity at 1:1 and 0.25:1 E:T (**A**), cell proliferation after 72 h at E:T 1:1 (**B**), and proinflammatory cytokine release after 24 h at E:T 1:3 (**C**). BLI tumor growth signal of NSG mice s.c. injected with 5 × 10^6^ luc+ HD-LM2 cHL cells on the right flank, treated at day +15 either with NT T cells or clone 142-derived anti-CD30 CAR-T cells (**D**). BLI signal from day +65, at tumor re-challenge in mice treated with anti-CD30 CAR-T cells receiving a second s.c. injection on the opposite flank together with 4 control mice. * *p* ≤ 0.05, ** *p* ≤ 0.01, *** *p* ≤ 0.001, **** *p* ≤ 0.0001 (**E**).

Anti-CD30 CAR-T and non-transduced T cells as a control were co-cultured to explore their cytolytic capacity against the HD-LM2 cell line at two different E:T ratios. At a 1:1 E:T ratio after 4 h co-culture, all anti-CD30 CAR-T cells strongly induced cell lysis of HD-LM2 cells compared to control cells. However, at a low and disadvantageous 0.25:1 E:T ratio, only clone 142-derived CD30 CAR-T cells significantly killed HD-LM2 cells (Figure 7A). To estimate CD30 CAR-T cell proliferation upon tumor challenge, we then co-cultured anti-CD30 CAR-T and control T cells with irradiated HD-LM2 for 72 h at E:T ratio 1:1. Clone 142-derived CD30 CAR-T cells showed a higher proliferation rate compared to the other clones, in line with cytotoxicity results (Figure 7B). Furthermore, the release of the main pro-inflammatory cytokines was assessed upon 24 h of co-culture with HD-LM2 at a 1:3 E:T ratio. Clone 142-derived CD30 CAR-T cells also displayed higher effector functions in terms of GM-CSF, IL-2, IFN-γ, and TNF-α production as compared to the other CD30 CARs and non-transduced T cells (Figure 7C).

The anti-tumor activity of clone 142-derived anti-CD30 CAR-T cells was further confirmed in vivo by a xenograft NSG mouse model of luciferase-positive HD-LM2 cells subcutaneous (s.c.) injection. A single infusion of anti-CD30 CAR-T cells after 15 days from tumor injection led to complete and stable disease eradication compared to control NT T cells (Figure 7D). Moreover, to further investigate clone 142-derived anti-CD30 CAR-T cells persistence capacity, previously treated mice were re-challenged with 5 × 10^6^ HD-LM2 LUC+ on the other flank, together with a four-mice control group. After three weeks, all control mice showed bioluminescence rapid tumor growth, while the clone 142-derived anti-CD30 CAR-T cells efficiently inhibited tumor engraftment, sustaining a complete remission from the first tumor injection due to prolonged CAR-mediated immune memory (Figure 7E).

According to in vitro assays, these in vivo data strongly sustain the anti-tumor activity exerted by clone 142-derived anti-CD30 CAR-T cells and further sustain the consistency of reported in silico predictions.

## 3. Discussion

Integrating recent advancements in machine learning with molecular docking and MD simulations may enhance the predictive power of molecular modeling in drug discovery. Since the interplay between monoclonal antibodies (mAbs) and target antigens relies on the structural and chemical complementarity between the antigen’s epitope and the single-chain variable fragment’s (scFv) paratope binding site [20], MD simulations could play a crucial role in modeling these interactions, calculating the motions and interfaces of all distinct atoms over time [21].

In this study, we present a rapid and cost-effective computational approach utilizing AI-driven molecular docking and MD simulations for the evaluation of three anti-CD30 mAbs, as an alternative approach for optimal scFv-based CAR development. Notably, reducing costs for research and development is one of the main challenges to decreasing the CAR-T cell price, typically over USD 400,000 per patient [22]. To validate our approach, we performed a direct comparison with SPR and functional in vitro and in vivo validation of scFv-derived CAR.

To confirm the reliability of our affinity ranking approach, we performed a direct comparison with surface plasmon resonance (SPR) and functional validation assays, both in vitro and in vivo, of single-chain variable fragment (scFv)-derived chimeric antigen receptors (CARs).

We revealed that MD simulations offer a realistic scFv characterization in terms of energetics and kinetics, assisting in the design of new CAR constructs. Although SPR is the best rapid and cost-effective high throughput screening of fragment libraries [23], it can be performed only when certain amounts of mAbs and target antigens are provided. Rigorous SMD may provide a surrogate of the association/dissociation rate constants through the estimation of binding free energy differences (∆G) among scFv–antigen complexes. Unlike the constrained nature of force–time curves from SMD, umbrella sampling simulations present a refined methodology. By systematically sampling a range of conformations along the dissociation pathway, umbrella sampling captures the complex energy landscape more comprehensively. Thus, we decided to use umbrella sampling simulations to calculate and compare ∆G of different anti-CD30 scFv binding. Our study confirmed that the higher computational power of the umbrella sampling simulations, over standard in silico methods in providing accurate results, has the potential to become an advanced and practical tool to investigate scFv affinity beyond SPR.

Moreover, AI-assisted MD simulations have the opportunity to accurately compare different scFvs given only their amino acidic sequences and, more importantly, to uncover key interactions between scFv and target antigens involved in binding [24]. Such a level of understanding may inform computational strategies to tune scFv affinity by site-specific mutagenesis of specific amino acids responsible for interaction strength. We have already shown the effectiveness of MD analysis in identifying low-affinity clones targeting CD123, minimizing off-target effects on healthy tissues expressing low levels of CD123 while retaining anti-tumor activity against CD123 highly positive acute myeloid leukemia cells in a dual-CAR transignalling model [25]. Knowledge of mAb-derived scFv binding interactions could be therefore essential to drive CAR immune functions, especially for target antigens co-expressed by tumor and healthy cells [26]. In this view, optimizing existing CDR sequences by AI-powered molecular docking and steered molecular dynamics could also be exploited to replace the generation of novel murine anti-human mAbs.

As we move toward a future marked by precision and efficiency in immunotherapies, the synergy between AI and molecular dynamics simulations holds great promise in accelerating the development of next-generation CAR-T cell therapies. This study proves how the combination of AI-driven data and physics-based simulations holds exciting potential to further improve other insights into CAR design, such as the identification of optimal scFv orientation or finest linker and spacer length, without the need for extensive functional comparisons between different CAR-redirected cell products.

## 4. Materials and Methods

### 4.1. Generation of Anti-CD30 Monoclonal Antibodies and scFv Sequencing

The anti-CD30 mAbs were produced at the academic Centro Ricerche Onco-Ematologico (CREO, Perugia, Italy). Briefly, recombinant peptide from the extracellular portion of CD30 (200 ng) was resuspended in PBS and administered intraperitoneally into Balb/c mice. After the fourth administration, mice were sacrificed and their spleens were used to generate hybridomas. Heavy- and light-chain variable domains sequencing of CD30 mAbs were obtained in outsourcing (Genescript, Piscataway, NJ, USA) after total RNA extraction from hybridoma cells, reverse transcription into cDNA.

### 4.2. Artificial Intelligence 3D Structure Prediction of the Studied Systems’ Antibody–Antigen

AlphaFold2 [13], a cutting-edge artificial intelligence (AI) system, was utilized to predict the 3D structures of proteins based on their amino acid sequences. To assess the quality and confidence of these predictions, we relied on the predicted local distance difference test (pLDDT) scores provided by the Colab-AlphaFold2 server [13]

To further validate the predicted protein structures, we employed additional assessment methods and incorporated experimental data, including two web servers: ERRAT and the Ramachandran plot from the Protein Structure Analysis and Verification Server (SAVE v6.0, accessible at https://saves.mbi.ucla.edu/ accessed on 10 June 2024).

The refinement of protein 3D models enhances the accuracy and quality of initial prediction results by employing optimization techniques that improve various aspects of the 3D structure (optimizing side-chain placement, resolving clashes and distortions, and refining both local and global structures). Based on the results presented in Supplementary Table S2 (Ramachandran plot) and Supplementary Table S3 (ERRAT scores), 3D structures of clones 231 and 142 improved after refinement, respectively from 93.69 to 95.14 and from 97.25 to 99.09 for quality factor. No improvement for clone Ber-H2 3D structure was shown after refinement as compared to initial values (95.41 quality factor).

This process yielded 5 predicted models for each provided sequence. To determine the optimal structure within each group for subsequent molecular docking, these models were further submitted to SAVE v6.0.

### 4.3. Artificial Intelligence-Driven Information Molecular Docking

Starting from the predicted antibody structure retrieved from Colab-AlphaFold2, our initial step involved identifying residues located within the hypervariable loops. These residues are crucial for steering the docking process, as previously elucidated by Ambrosetti et al. [27]. To identify these residues, we harnessed ProABC-2 antibody paratope prediction, a random-forest predictor underpinned by a convolutional neural network algorithm [28]. Following, the heavy- and light-chain sequences were submitted to the web server and the hypervariable loops were annotated. CD30 structure was obtained from AlphaFold2 and submitted to the DiscoTope-3.0 web server [29]. This particular server employs an XGBoost model to predict the epitope propensity on a per-residue basis. For the docking of antigen and antibody constructs, we turned to the HADDOCK [30] web server (accessible via https://wenmr.science.uu.nl/haddock2.4 accessed on 10 June 2024). The structure files of both antigens and antibodies were uploaded to the webserver where we precisely specified the active residues within both antigen and antibody constructs, which were predicted as previously described. The top 3 clusters of each docking complex were described.

### 4.4. All-Atom (AA) Molecular Dynamics (MD) Simulations

AA-MD simulations were performed using GROMACS v2022.5. All complexes were modeled with the Amber ff14SB [31] all-atom force field, embedded in a periodic cubic simulation box with dimensions chosen to provide a minimum 30 Å buffer of solvent molecules surrounding the solute, hydrated with a TIP3P [32] water model, and neutralized with a 0.15 M NaCl solution. Prior to the MD simulations, all systems underwent an energy minimization step involving 10,000 iterations of the steepest descents to relax the system and remove any steric clashes. The equilibration of each complex was carried out in two stages. During the first phase, position restraints were applied to heavy atoms, and the system was simulated for 500 ps under the constant volume (NVT) ensemble. In this phase, the temperature was maintained at 310 K using the V-rescale algorithms (Berendsen-modified weak coupling method) [33]. Subsequent to NVT equilibration, another 500 ps of equilibration was performed under constant pressure (NPT) conditions, utilizing Parrinello–Rahman algorithms [34] to maintain pressure isotropically at 1.0 bar.

After equilibration, completely unrestrained MD simulations were performed for 100 ns. For this data collection period, the V-rescale thermostat and the Parrinello–Rahman barostat were used to maintain the temperature at 310 K and isotropically regulate pressure at 1.0 bar, respectively. Long-range electrostatic interactions were calculated using particle mesh Ewald [35] with a cutoff of 1.2 nm, and a 1.2 nm cutoff was used for van der Waals interactions (the non-bonded Lennard–Jones terms). Structures from the end of each of these trajectories (142-CD30, 231-CD30, and BERH2-CD30) were used as starting configurations for pulling simulations.

### 4.5. Coarse-Grained (CG) Steered Molecular Dynamics (SMD)

Coarse-grained simulations for all systems were performed employing the SIRAH force field [36], which can be accessed at http://www.sirahff.com accessed on 10 June 2024. CG-SMD simulations provide a powerful toolset for accelerating the design and optimization of novel molecules by striking a balance between computational efficiency and the ability to extract meaningful insights into binding mechanisms and potential clinical behavior. The structures were placed in a rectangular box with dimensions sufficient to satisfy the minimum image convention and provide space for pulling simulations to take place along the XYZ-axis. WT4 water model [37], included in the SIRAH CG force field, was used to represent solvent, and 150 mM NaCl was also added to the simulation cell. All pulling simulations were conducted with the GROMACS v2022.5 package. Each system was then minimized in two stages. First, using the steepest descent for 10,000 steps energy minimization of side chains by restraining the backbone and then using the steepest descent for 10,000 steps energy minimization of the whole system. The equilibration of each complex was carried out in two stages. In the first stage, the system was simulated for 500 ps under the constant volume (NVT) ensemble and the temperature was maintained at 310 K using the V-rescale algorithms. During this stage, a 1000 kj mol^−1^ force position restraint was applied to heavy atoms, to perform solvent equilibration. In the next stage, soft equilibration to improve side chain solvation was performed. In this stage, a 100 kj mol^−1^ force position restraint was applied to heavy atoms, and another 500 ps of equilibration was performed under constant pressure (NPT) conditions, utilizing Parrinello–Rahman algorithms to maintain pressure isotropically at 1.0 bar.

After the equilibration process, restraints were removed from all antibodies except from the antigen (Figure 4). The antigen served as a fixed reference point in the pulling simulations. The application of position restraints is a common practice in simulations to emulate stability. In this case, it prevents the unfolding of the antigen and also avoids the antigen drifting with the antibody during the pull process. For each of the complexes, the antibody was pulled away from the core structure along the XYZ-axis (Figure 4) over 5 ns, using a spring constant of 1000 kJ mol^−1^ nm^−2^ and a pull rate of 1 nm ns^−1^ (10 Å ns^−1^). All the simulations used a time step of 20 fs, the particle mesh Ewald with a direct cutoff of 1.2 nm and a grid spacing of 0.25 nm, and a 1.2 nm cutoff for van der Waals (vdW) interactions. A final center-of-mass (COM) distance between the antibody and antigen of approximately 5.5 nm was achieved.

### 4.6. Coarse-Grained (CG) Umbrella Sampling (US) Simulation and Analysis of Binding Free Energy

From the SMD trajectories, snapshots were taken to generate the starting configurations for the umbrella sampling [38] windows. This ensures adequate sampling and convergence of the potential of mean force (PMF) calculations along the reaction coordinate, providing a detailed understanding of the energy landscape associated with the unbinding process. For each complex, a symmetric distribution of sampling windows was used, and the reaction coordinate for the umbrella sampling was discretized with a spacing of 0.05 nm up to 5.5 nm COM separation. Such spacing ensured sufficient overlap of the probability distribution of each window, and resulted in about 70 windows. In each window, minimization (10,000 steepest descent) and an NPT ensemble equilibration (1 ns) were performed using the same methodology described above, and 10 ns of MD was performed for umbrella sampling. We used the weighted histogram analysis method (WHAM) [39] to analyze the result and to reconstruct the PMF from US simulations [40]. For each complex, the binding free energy calculation (ΔG) was calculated from the PMF by taking the difference in free energy in the bound and unbound states.

### 4.7. Surface Plasmon Resonance (SPR)

SPR analysis was carried out with the ProteOn XPR36 Protein Interaction Array system (Bio-Rad, Hercules, CA, USA). The system allows up to six ligands to be immobilized on parallel lanes of the same sensor surface (including an “empty” or reference channel). The flow channels can be rotated 90° so that up to six analyte solutions can flow in parallel on all the immobilized ligands [41]. For these studies, we used the protocol described by Canziani et al., 2004 [42], using anti-mouse IgGs to capture the different antibodies from crude hybridoma samples on different lanes of the chip. After rotation of the flow channels, different concentrations of the antigen (CD30) could be flowed simultaneously on all the immobilized antibodies, allowing a reliable determination of the binding constants. Notably, the analysis of the binding of flowing CD30 to immobilized anti-CD30 mAbs avoids the avidity effects associated with the reverse design (flow of bivalent antibodies over immobilized antigen). Anti-mouse IgGs (AffiniPure Goat Anti-Mouse IgG, Fcγ Fragment Specific Jackson ImmunoResearch, Code n.: 115-005-008, Lot: 146,974) were immobilized in five parallel lanes of a CMD 700L sensor chip (XanTec bioanalytics), using amine coupling chemistry, as previously described [43]. Briefly, to activate the chip surface we injected a solution composed of 50 mM N-hydroxysuccinimide and 400 mM 1-ethyl-3-(3-dimethylaminopropyl)-carbodiimide (NHS/EDC), for 5 min at 30 µL/min. Then, the anti-mouse IgGs were injected for 5 min at 30 µL/min, at the concentration of 30 µg/mL in sodium-acetate pH 5.0 (NaOAc). The remaining activated carboxyl groups were deactivated by injecting 1 M ethanolamine for 5 min at 30 µL/min. Immobilization levels were about 5000 resonance units (RU, where 1000 RU = 1 ng protein/mm^2^), in the five lanes. The sixth lane remained empty, i.e., activated and deactivated but without the anti-Fc mAbs. Then, 3 different crude hybridoma samples, diluted 1:20 in SPR running buffer (Dulbecco’s Phosphate-Buffered Saline with 0.005% Tween-20, PBST) were flowed in four parallel lanes over pre-immobilized anti-mouse IgGs. Each sample was introduced twice for two min at a flow rate of 30 µL/min. The different mAbs were captured by the immobilized anti-mouse IgGs at very similar levels, ranging consistently between 878 and 978 RU, and the binding was highly stable. One lane was left with anti-mouse IgGs only, to be used as a reference surface.

After chip rotation, CD30 was injected at five different concentrations (1, 3, 10, 30, and 100 nM), simultaneously over all the captured mAbs, or anti-mouse IgGs or the empty surface, for 5 min at a rate of 30 μL/min. Dissociation was measured in the following 5 min. All assays were performed at 25 °C. The sensorgrams, representing the time course of the SPR signal in RU, were normalized to a baseline of zero. The specific CD30 binding to pre-captured mAbs was obtained by subtracting the non-specific response measured on the reference surface (anti-mouse IgGs only). The binding constants for each CD30–mAb interaction were obtained from simultaneous (global) fitting of the entire sensorgrams, including both association and dissociation phases, across all the CD30 concentrations. Sensorgrams were fitted using the simple 1:1 Langmuir model to obtain association and dissociation rate constants (ka and kd), equilibrium dissociation constant (KD), and maximum binding capacity (Rmax).

### 4.8. Generation of Anti-CD30 CAR-T Cells

Leukapheresis products were obtained from buffy coats of healthy donors following the acquisition of informed consent. Peripheral blood mononuclear cells (PBMC) were isolated through gradient centrifugation using Ficoll-Paque™ PLUS (Cytiva, Marlborough, MA, USA) followed by multiple washes in PBS containing EDTA 0,5M pH 8 and HSA 20%. T lymphocytes were enriched using anti-CD4 and anti-CD8 microbeads (Miltenyi Biotech, Santa Barbara, CA, USA) and LS separation columns (Miltenyi Biotech, Santa Barbara, CA, USA) following the manufacturer’s instructions. The isolated T cells were finally resuspended in a 24-well tissue culture treated at the concentration of 1 million/mL and activated for 24 h with 10 µL for each 1 million cells using T cell TransAct, a polymeric nanomatrix conjugated to humanized CD3 and CD28 (Miltenyi Biotech, Santa Barbara, CA, USA).

Activated T lymphocytes were transduced with PLVX-EF1α-IRES bicistronic lentiviral vector encoding the gene of interest, a second-generation CD28. CAR differing only for scFv (either derived from clone 142, 231, or BER-H2) fused to the ZsGreen reporter gene. Newly generated CAR-T cells were washed after 72 h from lentiviral infection (37 °C with 5% CO_2_) and expanded for 11 days in TexMACS™ Medium (Miltenyi Biotech, Santa Barbara, CA, USA) supplemented with 10 ng/mL of human IL-7 and 10 ng/mL of human IL-15 (Miltenyi Biotech, Santa Barbara, CA, USA). Before cytotoxicity, proliferation, and cytokine release assays, cell sorting of all CAR-T cell populations was performed to obtain over 95% comparable levels of ZsGreen CAR+ cells.

### 4.9. In Vitro and In Vivo Efficacy Evaluation of Anti-CD30 CAR-T Cells

Hodgkin lymphoma HD-LM2 cell line was obtained from the American Type Culture Collection. Cancer cells were cultured in RPMI 1640 medium supplemented with 20% fetal bovine serum (Gibco), 1% penicillin/streptomycin, and 1% glutamine, and incubated at 37° with 5% CO_2_. For the in vivo evaluation, HD-LM2 cells were engineered to stably express the luciferase gene in order to monitor tumor growth with bioluminescence imaging.

For the cytotoxicity assay, CAR-T cells or untransduced T cells were co-cultured in triplicate in a 96 well with the Hodgkin lymphoma cell line HD-LM2 at the different effector:target (E:T) ratio of 1:1 and 0.25:1. After 4 h of incubation (37 °C with 5% CO_2_), cells were collected, washed with 1 mL PBS BSA 2% (2000 rpm × 3 min), and stained (4 °C, 15 min at dark) with (APC)-ANNEXIN-V, 7-AAD and (PE)-anti-CD30 antibodies. After a second wash with 1 mL PBS BSA 2% (2000 rpm × 3 min), samples were analyzed by flow cytometry.

To investigate CD30 CAR-T cell proliferation, co-culture between anti-CD30 CAR-T cells or untransduced T cells and HD-LM2 were performed at E:T ratio of 1:1. The Hodgkin lymphoma HD-LM2 cell line was previously gamma-irradiated in order to block the autologous ability to proliferate and then plated in triplicate in a 96 well (U bottom, tissue culture treated) and harvested after 72 h (37 °C with 5% CO_2_) to be analyzed with Click-iT™ EdU Cell Proliferation Kit (Thermo Fisher, Waltham, MA, USA), following the manufacturer instructions.

To measure the concentration of the main pro-inflammatory cytokines, CD30 CAR-T cells or untransduced T cells were co-cultured with HD-LM2 cell line at E:T ratio of 1:3. Cells were plated in triplicate in a 96 well (U bottom, tissue culture treated) and, after 24 h (37° with 5% CO_2_), they were harvested and centrifuged at 1000× *g* 10 min 4 °C. Supernatants were then collected to be analyzed with MACSPlex Cytokine 12 kit, human (Miltenyi) following the manufacturer instructions. Then, 8–12-week-old non-irradiated NSG mice were subcutaneously (s.c.) injected in the right flank with 5 × 10^6^ HD-LM2 luciferase positive cells. After 15 days from injection, mice were intravenously inoculated with 2.5 × 10^6^ NT T cells or clone 142-derived anti-CD30 CAR-T cells. The two groups of mice were monitored weekly by bioluminescence imaging using IVIS Lumina III (Perkin Elmer, Waltham, MA, USA). At day 65, mice were tumor re-challenged with 5 × 10^6^ HD-LM2 LUC+ s.c. injected in the left flank and monitored weekly.

### 4.10. Statistical Analysis

Results are reported as mean ± standard deviation (SD) of three independent experiments. Statistical significance was determined by two-way ANOVA carried out by Prism v. 8.0.1.224 (GraphPad Software, La Jolla, CA, USA), or repeated measures analysis of variance followed by Dunnett’s test for multiple comparisons performed in R (R version 4.3.1, reference. R Core Team (2023). _R: A Language and Environment for Statistical Computing_. R Foundation for Statistical Computing, Vienna, Austria. <https://www.R-project.org/> accessed on 10 June 2024). *p* * <  0.05 was considered as significant.

## Data Availability

All data supporting the conclusions of this study are provided within figures, the table, Supplemental Information, and Supplemental Tables. Raw data files can be provided by the corresponding authors, F.M. and V.M.P., upon reasonable request.

## Supplementary Materials

The following supporting information can be downloaded at: https://www.mdpi.com/article/10.3390/ijms25137231/s1.
