# Supplementary material for: Artificial Intelligence-Powered Molecular Docking and Steered Molecular Dynamics for Accurate scFv Selection of Anti-CD30 Chimeric Antigen Receptors
Source: Int J Mol Sci. 2024 Jun 30;25(13):7231. doi: 10.3390/ijms25137231 (PMC11242627; doi:10.3390/ijms25137231)
Supplement: Supplementary file 1 [file ijms-25-07231-s001.zip › ijms-3079466-supplementary.pdf]

Supplementary Table S1. HADDOCK AI-Information-Driven Docking results of the 3 best clusters for the CD30 F19-P154 and CD30 T230-A307 regions.

Complex	HS[1] Cluster1 (HB[2])	HS[1] Cluster2 (HB[2])	HS[1] Cluster3 (HB[2])
142–CD30 $^{F19\text{-}P154}$	-98.3 ± 3.8 (13)	-87.4 ± 3.6 (12)	-83.6 ± 3.0 (11)
142–CD30 $^{T230\text{-}A307}$	-89.6 ± 4.5 (10)	-77.2 ± 2.9 (10)	-68.6 ± 5.6 (11)
231–CD30 $^{F19\text{-}P154}$	-98.0 ± 3.3 (13)	-83.2 ± 5.0 (7)	-79.1 ± 3.6 (8)
231–CD30 $^{T230\text{-}A307}$	-79.3 ± 3.3 (11)	-70.8 ± 0.6 (5)	-55.3 ± 3.4 (6)
Ber-H2–CD30 $^{F19\text{-}P154}$	-71.2 ± 4.2 (11)	-69.4 ± 4.7 (7)	-66.2 ± 4.8 (9)
Ber-H2–CD30 $^{T230\text{-}A307}$	-61.2 ± 4.6 (11)	-58.9 ± 1.0 (11)	-51.1 ± 4.2 (11)

[1] HS: HADDOCK score
[2] HB: Hydrogen Bonds number

Supplementary Table S2. Ramachandran plot results before and after refinement.

Ab clones	Before Favored regions	Before Allowed regions	Before Disallowed regions	After Favored regions	After Allowed regions	After Disallowed regions
142	91.1%	8.9%	0.0%	93.1%	6.9%	0.0%
231	89.5%	9.5%	1.1%	87.4%	11.6%	1.1%
BerH2	89.09%	8.5%	1.5%	88.09%	10.0%	1.0%

The Ramachandran plot was used to examine the angles of amino acids within the protein's backbone. Favored regions, encompassing angles that are highly energetically favorable, correspond to stable secondary structures like alpha helices and beta sheets. Allowed regions consist of angles that are sterically allowed but possess lower energetic favorability. Disallowed regions, encompassing angles that are either sterically or energetically unfavorable, potentially resulting in structural problems or instability within the protein.

Supplementary Table S3. ERRAT scores and per-residue plot before and after refinement.

Ab clones	Before refinement	After refinement
	**Overall quality factor**	
142	97.25	99.09
231	93.69	95.14
BerH2	95.41	92.23
	**per-residue plot**	
142	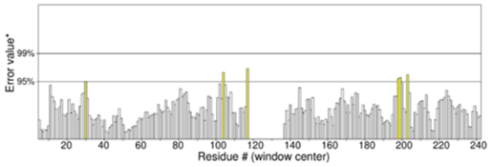	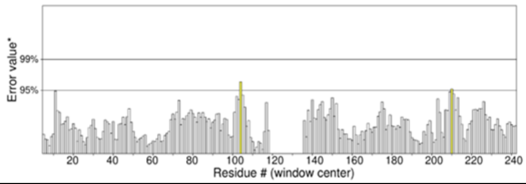

231	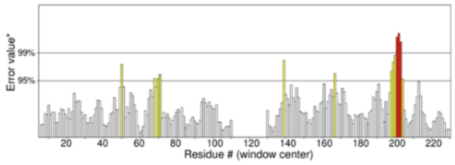	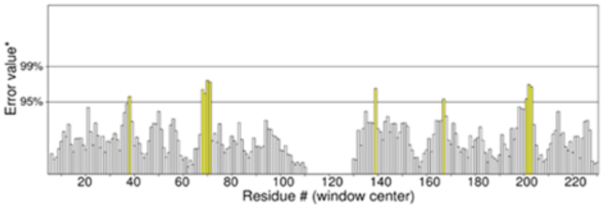
BerH2	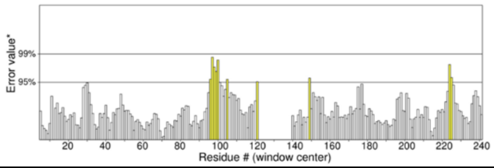	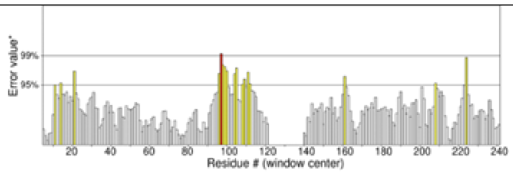

The ERRAT tool analyzed nonbonded atom interactions and compared them to high-resolution crystallography statistics. High scores indicate superior quality of protein structure, while lower scores indicated potential errors or inaccuracies. ERRAT per-residue plot highlight regions with low scores to identify potential errors or inaccuracies in specific areas.
